# Nanoscale diffusion in the synaptic cleft and beyond measured with time-resolved fluorescence anisotropy imaging

**DOI:** 10.1038/srep42022

**Published:** 2017-02-09

**Authors:** Kaiyu Zheng, Thomas P. Jensen, Leonid P. Savtchenko, James A. Levitt, Klaus Suhling, Dmitri A. Rusakov

**Affiliations:** 1 Institute of Neurology, University College London, Queen Square, London WC1N 3BG, UK; 2 Institute of Neuroscience, University of Nizhny Novgorod, 603950 Nizhny Novgorod, Russia; 3 Department of Physics, King’s College London, Strand, London WC2R 2LS, UK

## Abstract

Neural activity relies on molecular diffusion within nanoscopic spaces outside and inside nerve cells, such as synaptic clefts or dendritic spines. Measuring diffusion on this small scale *in situ* has not hitherto been possible, yet this knowledge is critical for understanding the dynamics of molecular events and electric currents that shape physiological signals throughout the brain. Here we advance time-resolved fluorescence anisotropy imaging combined with two-photon excitation microscopy to map nanoscale diffusivity in *ex vivo* brain slices. We find that in the brain interstitial gaps small molecules move on average ~30% slower than in a free medium whereas inside neuronal dendrites this retardation is ~70%. In the synaptic cleft free nanodiffusion is decelerated by ~46%. These quantities provide previously unattainable basic constrains for the receptor actions of released neurotransmitters, the electrical conductance of the brain interstitial space and the limiting rate of molecular interactions or conformational changes in the synaptic microenvironment.

Rapid chemical signalling in the CNS relies on free molecular diffusion in the interstitial space. The efforts to gauge brain extracellular diffusivity have progressed from using radiotracers^1^ and iontophoretic current measurements^2^ to the point-source fluorescence imaging^3^,^4^,^5^ which can be further refined using micro fibre optics^6^. However, the scale of such measurements (>2–3 μm) necessarily incorporates space tortuosity (geometric hindrance) arising from microscopic obstacles pervading the neuropil. How fast the small signalling molecules move on the nanoscale in the brain has thus remained an enigma. Resolving this issue is nonetheless important, for several demonstrable reasons. First, nanoscale mobility of free-diffusing molecules and ions determines electric conductance of the medium and thus directly guides our knowledge about local electric currents pertinent to neural activity. Second, this mobility sets the maximum rate for local diffusion-limited biochemical reactions. Finally, it determines the extent to which synaptically discharged neurotransmitters or other signalling molecules activate synaptic and extra-synaptic receptors, a long-standing question in neurobiology^7^,^8^,^9^,^10^,^11^,^12^,^13^.

One photonics technique that can evaluate ‘instantaneous’ mobility of fluorescent molecules is termed time-resolved fluorescence anisotropy imaging (TR-FAIM)^14^,^15^,^16^. This method exploits the fact that the fluorescence emitted by fluorophores is polarised, and only the fluorophore molecules with their transition dipole moment aligned with the polarisation plane of excitation are excited. Because fluorescent excitation and emission are normally separated by 0.1–10 ns, the polarization plane of emission diverges from that of excitation due to Brownian motion, i.e. depending on how fast the molecule moves and gyrates in space (Fig. 1a). Thus, when molecules are excited by polarised light, their mobility can be mechanistically related to the time-resolved fluorescence anisotropy decay (Fig. 1b, Supplementary Fig. S1a,b). This method is independent of the fluorophore concentration, which is difficult to control in tissue.

We have earlier developed a TR-FAIM approach that enables high-resolution mapping of intracellular diffusivity in relatively translucent, optically homogenous cell cultures^17^,^18^,^19^. However, intact animal tissue could be a highly turbid, optically heterogeneous medium which requires a dedicated set of methods for successful implementation of TR-FAIM^20^. In addition, high-resolution fluorescence imaging in organised tissue usually requires a femtosecond-pulse infra-red laser, to ensure highly confocal two-photon excitation (2PE) by ballistic photons, with no concomitant light scattering^21^. Bearing this in mind, we therefore attempted to develop an approach that would enable the mapping of nanoscale molecular diffusivity in the mammalian brain tissue, inside and outside nerve cell compartments, in the established preparation of acute hippocampal slices under full electrophysiological control.

## Results

### Rotational versus translation diffusion

**Figure 1:**
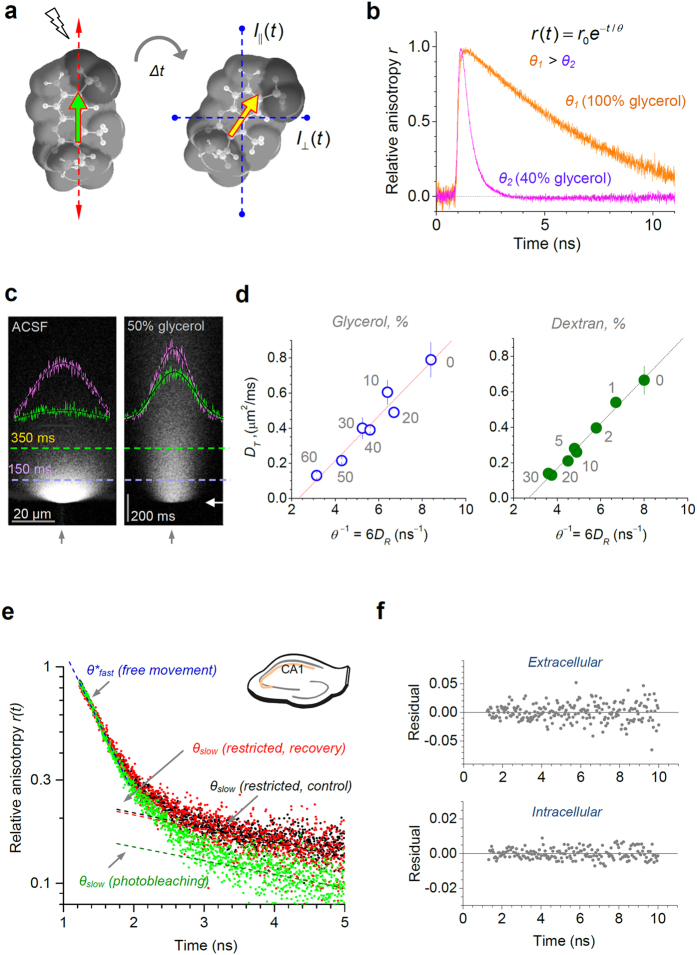
TR-FAIM readout to gauge molecular diffusion on the nanoscale. (**a**) Alexa Fluor 350 (AF350, 3D-diagram with Chem3D-Ultra) excited with polarised light (left: red arrows, polarisation plane; green arrow, excitation plane) moving and rotating over time *Δt* before emitting in the original excitation plane (yellow arrow); emission recorded through analysers *I*_P_(*t*) and *I*_⊥_(*t*). (**b**) Example: AF350 fluorescence anisotropy time course 
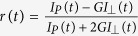
 (Methods), in 40% (less viscous) and 100% (more viscous) aqueous glycerol solution, as indicated; decay constant *θ* (‘rotational correlation time’) scales with viscosity; raw normalised data are shown including instrument response (Supplementary Fig. S1a,b). (**c**) AF350 translational diffusivity *D*_*T*_ measured independently of TR-FAIM: examples of line-scan 2PE images of a diffusion point-source (*x*-*y* position shown by grey and white arrowheads; 5 ms pulse, 1 μm-tip pipette) in ACSF (left) and 50% glycerol (right), as shown earlier^5^,^23^. Magenta and green traces, intensity profiles (proportional to extracellular dye concentration) at 150 ms and 350 ms post-pulse (dotted lines); black curves, best-fit for diffusion equation estimating *D*_*T*_, as described^5^,^23^. (**d**) *D*_*T*_ plotted against *θ*^−1^ (rotational diffusion coefficient; Methods) in aqueous solutions of varied viscosity: w/w percentage concentrations of glycerol (MW 92, left) and dextran (MW ~30 K, right) are shown; dotted lines, best-fit linear regression (Methods). (**e**) Separating the fast (major) and the slow (minor) components of the AF350 *r(t*) decay representing, respectively, free-moving and immobilised molecules in the slice extracellular space (hippocampal area CA1, inset). Black dots, control (4 min low laser power); green, photobleaching (10 min high power); red, recovery (low power): one experiment shown. Photobleaching selectively reduces the immobile fraction (slow decay component *θ*_*slow*_), without affecting the free-moving fraction (fast component 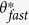). Data include instrument response (Fig. S1b; thus 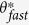 appears slower than the corrected *θ*_*fast*_, Table S1). (**f**) Characteristic (for >92% of all ROIs) residual plots for the double-exponent (*θ*_*slow*_, *θ*_*fast*_) fitting of the AF350 *r(t*) decay; symmetric Gaussian scatter indicates that additional exponential components are unlikely to be significant.

To understand diffusivity of small molecules in live brain tissue, we employed as a probe one of the smallest available cell-impermeable fluorescent indicators Alexa Fluor 350 (AF350, M.W.349), which has no lifetime dependency on physiological cellular environment and is entirely compatible with cell function^22^. In control experiments, we asked how the TR-FAIM-measured rotational diffusivity of AF350 *D*_*R*_ (which is inversely proportional to rotational correlation constant *θ*) is related to its translational diffusivity *D*_*T*_, over a range of medium viscosities. To measure *D*_*T*_ independently of TR-FAIM, we employed a point-source diffusion imaging technique implemented previously^5^,^23^ (Fig. 1c) and compared the outcome with the TR-FAIM readout in the same experiments. We found that *D*_*R*_ scaled nearly perfectly linearly with *D*_*T*_ values (Fig. 1d), in full accord with theory (Methods).

### Validating and calibrating TR-FAIM diffusion measurements in organised brain tissue

In this study, we focused on the common preparation of acute brain slices: we have previously found that this preparation shows excellent viability in compatible conditions of 2PE time-resolved fluorescence imaging combined with patch-clamp^24^. For the purpose of extracellular diffusion mapping, we added AF350 (0.5–1.0 mM) to the bath medium: this procedure appears fully consistent with normal neuronal activity and healthy slice environment^22^. Building upon our earlier explorations of TR-FAIM and related optical techniques^17^,^20^, we designed and carried out a series of control experiments aimed to validate and calibrate the method.

First, we found that the AF350 fluorescence anisotropy decay across slice tissue areas was best fitted using two exponential components, a major fast (decay constant 0.2–0.4 ns) and a 7–20 times smaller slow (2–12 ns) component, with only the fast component being present in a free bath medium (Fig. 1e,f; Supplementary Table S1, Supplementary Fig. S1c). The most straightforward explanation was that the two components represented AF350 fractions diffusing either freely (fast decay, >85% fraction) or in a restricted manner (slow decay, <15% fraction, likely due to residual binding of AF350 to cell membrane components). To validate this, we implemented a photobleaching test: unlike immobile AF350, the freely diffusing extracellular AF350 should not be sensitive to photobleaching because it is replenished almost instantaneously from an unlimited source (the surrounding bath medium). We found that photobleaching indeed dramatically reduced the slow-decaying anisotropy component while having little effect on the fast-decaying one (Fig. 1e), thus clearly associating the former with free extracellular diffusion. Consistent with this, washing out AF350 greatly increased the slow component of the anisotropy decay (Supplementary Fig. S1d).

**Figure 2:**
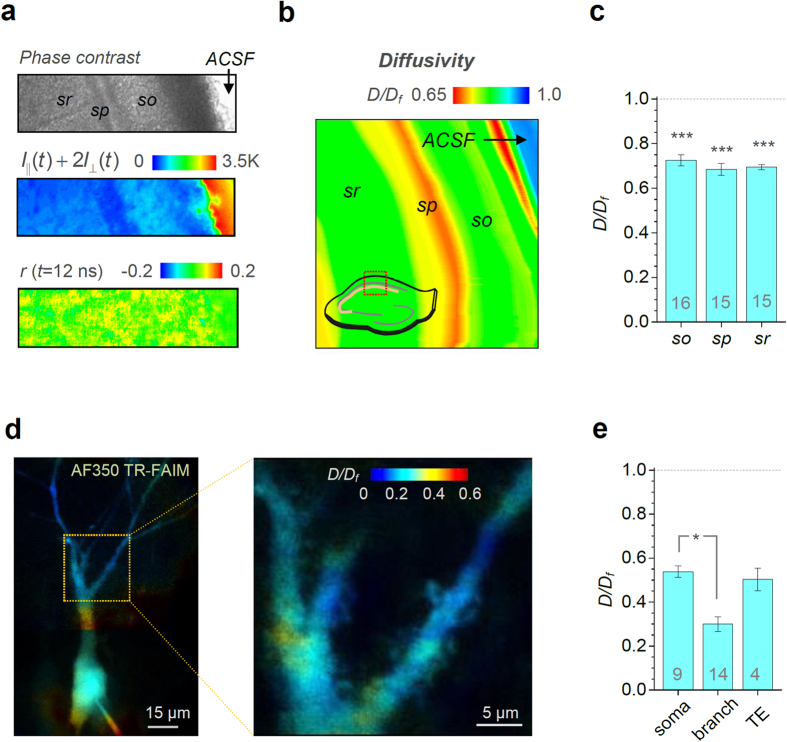
Mapping extracellular and intracellular molecular mobility in a hippocampal slice with TR-FAIM. (**a**) A fragment of hippocampal slice (*sr, s. radiatum*; *sp, s. pyrmidale*; *so, s. oriens*; *ACSF*, free medium area) depicted using phase contract (top, grey scale), total fluorescence intensity (middle; false colour scale), and an *r(t*) map at *t* = 12 ns post-pulse (bottom; ‘tail matching’ photonics control - condition of isotropic polarisation, or full depolarization); for control purposes, the inherent system’s anisotropy was isolated by comparing *I*_P_(*t*) and *I*_⊥_(*t*) in the bath medium at full isotropy (see *G*-factor in Methods). The homogeneous ‘tail-matching’ map (bottom) confirms that the TR-FAIM readout is not biased by uneven optical properties of the preparation, bath medium, or by the polarisation channel imbalance. (**b**) Map of the effective-to-free diffusion coefficient ratio *D/D*_*f*_ in the slice extracellular space (~100 μm deep, AF350) obtained by pixel-by-pixel calculation of *θ*; inset diagram, the area of interest (dotted rectangle) in the hippocampal slice. (**c**) Statistical summary of *D/D*_*f*_ values (mean ± SEM) averaged across the respective hippocampal areas, as in (**b**); numbers inside bars, sample size; ***p < 0.005 (*t*-test, compared to the free medium value). (**d**) TR-FAIM map of *D/D*_*f*_ inside a CA3 pyramidal cell loaded whole-cell with AF350, obtained at low (left, collage of two images) and high (right) magnification (zoomed area indicated). False colour *D/D*_*f*_ scale applies throughout. (**e**) Statistical summary of experiments shown in (**d**). *D/D*_*f*_ values (mean ± SEM) recorded from somata (*soma*), dendritic compartments (*branch*), and from inside the groups of thorny excrescences (TE) of CA3 pyramidal cells. Numbers inside bars, sample size; *p < 0.02.

Next, we established that any residual AF350 photobleaching during TR-FAIM could be readily accounted for (Supplementary Fig. S2a,c) and that no error was generated due to light scattering in the slice (Supplementary Fig. S2d). Finally, we determined that the concomitant fluorescent signal due to residual uptake of AF350 by cells in the slice did not exceed 4–6% (Supplementary Fig. S2e,f). Our further tests also verified that the highly uneven optical properties of the slice preparation did not bias the readout: the anisotropic (depolarised) fluorescence signal was homogeneously distributed, around its expected zero value, throughout the imaged field including both highly turbid tissue and highly translucent bath medium areas (Fig. 2a). Taken together, these observations indicated that the TR-FAIM technique was appropriate for mapping the mobility of free-diffusing AF350 across the slice tissue, with the known optical concomitants fully accounted for.

### Instantaneous extracellular and intracellular diffusivity in acute brain slices

We therefore carried out systematic measurements of extracellular diffusivity across area CA1 of acute hippocampal slices, routinely focusing the system at a depth of ~100 μm to ensure healthy conditions of local cells and tissue, as shown previously in similar settings using whole-cell recordings^24^. The TR-FAIM readout in our tests thus represented a pixel-by-pixel map of the effective-to-free diffusion coefficient ratio (*D*/*D*_*f*_, the local diffusion coefficient *in situ* relative to that in a free bath medium), for extracellular AF350 (one-slice example in Fig. 2b). A summary of such measurements (n = 16 slices) indicated that the area-average instantaneous extracellular molecular mobility was approximately 70% of that in a free medium (ACSF), with slight variations occurring among hippocampal regions (Fig. 2c). This result shows that small signalling molecules, including major ions of the extracellular medium, diffuse on the nanoscale on average ~30% slower in the brain interstitial space compared to a free cerebrospinal fluid.

With the TR-FAIM technique thus validated for optically heterogeneous slice tissue, we next sought to map instantaneous molecular mobility inside individual nerve cells. We held the principal neurons (CA3 pyramidal cells) in whole-cell mode loading them with AF350. The TR-FAIM mapping revealed heterogeneous landscapes of molecular diffusivity across cellular compartments (Fig. 2d,e), with an average *D*/*D*_*f*_ ranging from 0.30 ± 0.03 (n = 14) in dendritic branches, to 0.50 ± 0.05 (n = 4) inside postsynaptic spinous structures (classically termed ‘thorny excrescences’, TE; also see below), to 0.54 ± 0.03 (n = 9) in the soma. These results reveal that, firstly, rapid diffusion exchange and therefore diffusion-limited molecular reaction cascades should proceed, on average, two times slower inside cells than in a free medium. Secondly, these reaction limiting rates vary significantly among distinct neuronal compartments.

### Instantaneous diffusion inside the nanoscopic synaptic cleft

**Figure 3:**
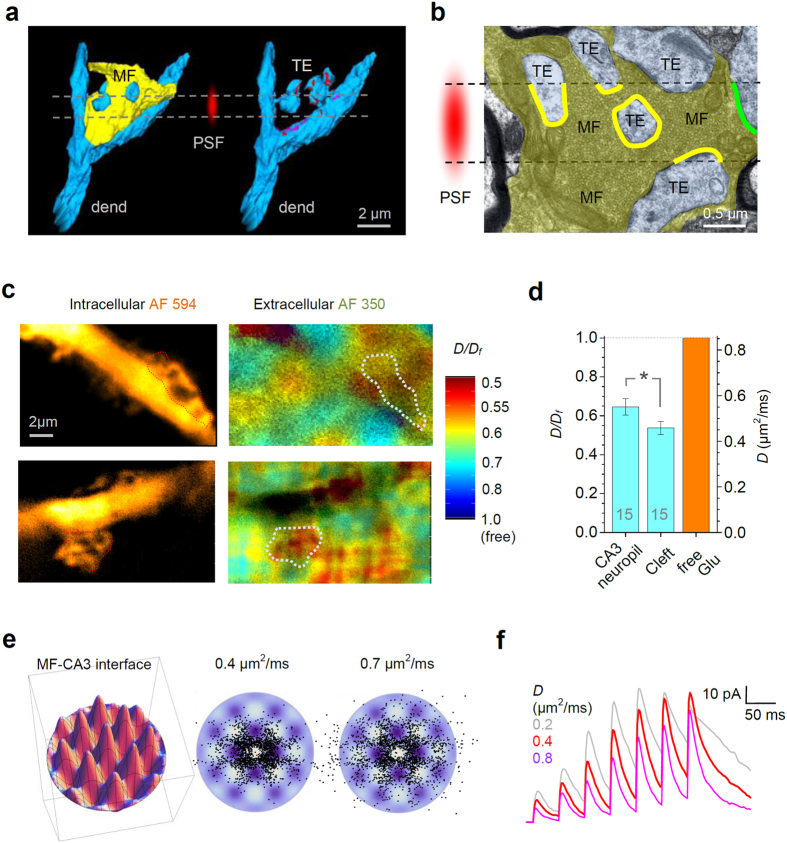
Molecular diffusivity inside and outside the synaptic cleft. (**a**) EM-based 3D reconstruction of the characteristic MF-CA3 synapse (modified from ref. 29) depicting a giant presynaptic bouton (yellow) and multiple postsynaptic dendritic spines (thorny excrescences, blue; red spots, individual postsynaptic densities). Red oval illustrates the characteristic 2PE point-spread function (PSF) which delimits the focal layer excitation (dotted lines). (**b**) A typical single EM section of MF-CA3 synapses, as illustrated in (**a**), with presynaptic (MF, dark yellow) and postsynaptic (TE, blue) fragments (modified from ref. 29), and a characteristic PSF (red oval). When the focal layer (dotted lines) crosses postsynaptic spines it will excite a fluorophore which occurs mainly in the space between the pre- and postsynaptic membranes (bright yellow segments). (**c**) Examples of intensity images (left, whole-cell AF594) and TR-FAIM diffusivity maps (right, extracellular AF350; false colour scale) recorded simultaneously in the focal plane of postsynaptic thorny excrescences in CA3 pyramidal cells (as in (**b**)). ROIs (dotted line-delimited areas) depict the area of identifiable TEs (left) over which TR-FAIM readout is collected for intra-cleft measurements. (**d**) Statistical summary, extracellular *D/D*_*f*_ values (mean ± SEM) recorded in CA3 neuropil and inside the TE area (*Cleft*), as indicated; green (right ordinate), diffusion coefficient of glutamate in ACSF, shown for reference. Numbers inside bars, sample size; *p < 0.02. (**e**) A Monte Carlo model of glutamate diffusion inside the typical tortuous cleft of MF-CA3 synapses. 3D diagram: the ‘average’ interface of pre- and postsynaptic membranes at MF-CA3 synapses which recapitulates characteristic synapse geometry^29^, as detailed earlier^31^. Diagrams in the middle: snapshot of the synapse interface (frontal projection) showing diffusing glutamate molecules at 1 ms post-release (2500 molecules) from the centre, at two values of the diffusion coefficient, as indicated. See Methods and ref. 31 for detailed model description. (**f**) Simulated NMDA receptor currents at MF-CA3 synapses at different values of the intra-cleft diffusion coefficient for glutamate (as indicated); simulation data recapitulate synaptic responses to a train of seven action potentials at 20 Hz, with multiple release sites and progressively increasing release probability^31^; all other parameters kept unchanged throughout.

How rapidly the signalling molecules (neurotransmitters and ions) move inside the synaptic cleft has been a subject of long-standing debates^8^,^9^,^10^,^25^. In our extracellular TR-FAIM measurements (Fig. 2b) the minimum averaging tissue volume is given by the system’s point-spread function (PSF), which in the present imaging conditions is expected to be approximately 0.3–0.5 μm wide and 1–1.5 μm high. Because common excitatory synapses (such as CA3-CA1 connections pervading CA1 neuropil) are only 200–300 nm wide^26^,^27^, such measurements represent mainly the extracellular space outside the synaptic clefts. We therefore turned to the giant synapses formed by hippocampal mossy fibres (MFs) on CA3 pyramidal cells. These connections comprise 4–8 μm wide MF axonal boutons that engulf 10–20 characteristic postsynaptic spines forming TEs on CA3 cell primary or secondary dendrites^28^,^29^ (Fig. 3a; see Fig. 2d depicting the characteristic postsynaptic cell and its TEs). Therefore, when a 2PE microscope system is focused near the middle of such synapses, it will excite almost exclusively the extracellular fluorophores occurring between presynaptic MF and postsynaptic TE membranes (bright yellow segments in Fig. 3b).

To enable such measurements, the region of interest has to be restricted to MF-CA3 connections. We therefore used two chromatically separable indicators, one in the extracellular space (AF350), and one (Alexa Fluor 594, AF594) loaded whole-cell to visualise postsynaptic thorny excrescences in CA3 pyramidal cells, as described in detail earlier^30^. This enabled the coordinated localization of the extracellular fluorescence (AF350 channel) originating from within the area of MF-CA3 connections, with TEs identified in the AF594 channel (Fig. 3c).

These tests arrived at an average *D*/*D*_*f*_ ratio of 0.54 ± 0.03 (n = 15) inside the synaptic apposition cleft. This value was lower (*p* < 0.02) than the average value of 0.65 ± 0.04 (n = 15) in the greater (>50 μm wide) CA3 neuropil area which includes mostly non-synaptic extracellular space (Fig. 3d). With the diffusion coefficient of glutamate in a free ACSF of ~0.86 μm^2^/ms (ref. 25), this data point to ~0.46 μm^2^/ms as glutamate diffusivity between pre- and postsynaptic membranes in organised brain tissue.

### Implications for our knowledge of synaptic transmission

To see whether and how our findings affect our knowledge about synaptic receptor activation inside and in the vicinity of active synapses, we explored a detailed 3D Monte Carlo model of the hippocampal MF-CA3 synapses examined here (Fig. 3c). The model which recapitulates key (average) geometrical features of these synapses (Fig. 3e) has recently been extensively tested and validated against experimental recordings involving genetic probing of NMDA receptor isoforms^31^. Our simulations have indicated that NMDA receptor-mediated synaptic currents at this synapse strongly depend on the (hitherto unknown) intra-cleft diffusion coefficient of glutamate even when all other modelled synaptic features remain unchanged (Fig. 3e). Thus the knowledge of the correct glutamate diffusivity has an immediate impact on our mechanistic interpretation of experimental recordings in this case. It has been a fortuitous coincidence that the diffusion coefficient value of 0.46 μm^2^/ms obtained here with TR-FAIM is close to the 0.4 μm^2^/ms value adopted in an earlier study^31^ and therefore does not challenge the conclusions therein.

In another recent study, an experiment-based assessment of vesicular glutamate content at cerebellar mossy fibre - granule cell synapses was carried out assuming that the glutamate diffusion coefficient could fall anywhere between 0.3–0.6 μm^2^/ms^32^ (Supplementary Fig. S3a). Again, the presently obtained diffusivity value (0.46 μm^2^/ms) constrains this estimate substantially, suggesting that the vesicular glutamate content at these central synapses is in the range of 1800–2200 molecules (Supplementary Fig. S3b). Further implications of the present findings, including a wider physiological context, are discussed below.

## Discussion

Here we have introduced and adapted a time-resolved fluorescence microscopy method (TR-FAIM) to map nanoscale diffusivity of small molecules in organised brain tissue, the task which has not hitherto been attainable. Equipped with this TR-FAIM approach, we have estimated how rapidly molecular diffusion proceeds in the brain interstitial space, inside neuronal compartments and between pre- and postsynaptic membranes. These measurements have potentially important consequences for our understanding of basic mechanisms pertinent to brain function.

A strong dependence between the assumed (unknown) glutamate diffusion coefficient and the theoretical extent of glutamate escape, hence plausible extra-synaptic activation of glutamate receptors, has long been appreciated in the context of common excitatory synapses such as hippocampal CA3-CA1 connections^33^,^34^,^35^. The glutamate diffusivity value obtained here (0.46 μm^2^/ms) is consistent with the physiological scenario recently simulated for the three-dimensional CA1 neuropil containing multiple, stochastically activated excitatory synapses^36^. In that scenario (which adopts extracellular glutamate diffusivity of 0.4 μm^2^/ms), NMDA receptor subunits outside active synapses do get bound (and eventually activated), to a significant degree, by the escaping glutamate during physiologically relevant synaptic network activity even though the volume-average concentration of extracellular glutamate remains negligible^36^. This spatiotemporal integration of excitatory neurotransmitter signals by NMDA receptors is consistent with earlier estimates of sustained NMDA receptor occupancy during physiological network activity^37^ and could explain observations suggesting that backpropagating action potentials in CA1 pyramidal cells activate the NMDA receptors which have been ‘pre-bound’ by glutamate escaping from some earlier activated synapses, be they on the given or a neighbouring cell^38^.

Whilst the presently estimated value of ~0.46 μm^2^/ms for intra-cleft glutamate diffusion is close to 0.4 μm^2^/ms adopted in some earlier studies^31^,^32^, it appears higher than an estimate of ~0.3 μm^2^/ms obtained for cerebellar mossy fibre - granule cell synapses based on electrophysiological tests^9^. This apparent discrepancy, however, could be explained by the longer-range space tortuosity (spanning across neighbouring synaptic sites) included in the latter estimate. At the same time, the present value is significantly lower than the free-medium diffusivity value which was suggested earlier to apply to the synaptic cleft^8^.

The consequences of the present findings in a wider physiological context also seem important. Firstly, the extracellular nanoscale diffusivity value measured here implies that the equivalent electrical resistance of the brain extracellular medium (excluding geometric hindrance), the parameter which is unfeasible to measure directly, is on average ~30% higher than that of a free ACSF. Given the resistance value for ACSF of ~59 Ω·cm^39^, the extracellular milieu has therefore electrical resistance of ~77 Ω·cm. This correction will constrain the range of local electric currents in the interstitial space also affecting directly our assessment of both cell excitability and action potential propagation in neurons, as demonstrated by simple tests with a biophysical (NEURON-built) compartmental model of a neuron (Supplementary Fig. S3c). Importantly, the heightened extracellular resistance also suggests that electrophysiological measurements obtained in isolated or cultured nerve cells (normally surrounded by a free bath medium) could consistently differ from those obtained from similar cells *in situ*. Furthermore, the architecture of the extracellular space - which involves elements of the extracellular matrix - can change in different physiological states or under some pathological conditions and thus affect the neuronal network activity resulting in changed higher brain function^40^,^41^. The role played by the altered extracellular diffusivity in such effects remains to be established^42^,^43^.

Secondly, extracellular micro-viscosity could, at least in theory, directly influence rapid conformational changes of protein domains. In the classical squid giant axon experiment, an experimental increase in the medium viscosity by 30–40% slows down the recorded gating time of sodium channels by more than two-fold^44^. Thus, extrapolating channel activation kinetics measured *in vitro* onto organised brain tissue should account for the increased extracellular nanoscale viscosity. Similarly, accurate estimates of diffusion-limited reaction rates *in situ* have to incorporate the slowdown factors reported here. Indeed, in the case of membrane protein reactions it has been demonstrated that the diffusion-controlled reaction rate constant scales with the mean square displacement (i.e. diffusion coefficient) of a receptor-ligand complex in the intracellular milieu^45^. Inside nerve and glial cells, nanoscale diffusivity is key to diffusion separation of individual microscopic cell compartments, such as dendritic spines in neurons^46^ or endfoot processes in astroglia^47^, as well as to diffusion-limited aggregation and polymerization of (aberrant) protein molecules associated with cell pathologies^48^. The knowledge about microscopic mobility of signalling molecules thus appears essential for our understanding of basic brain functions.

## Methods

### Two-photon TR-FAIM: imaging system

Two-photon excitation by short infrared laser pulses was used to restrict emission collection to a thin focal excitation plane ~100 μm deep into the slice. We thus ensured that no contaminating fluorescence was collected from damaged tissue near the slice surface (no detectable autofluorescence from the slices was recorded before applying AF350). The imaging system was based on Biorad Radiance 2000 (Zeiss) microscope, integrated with a SPC-830 TCSPC Becker & Hickl imaging module and a SpectraPhysics MaiTai laser, pulsing at 80 Mhz with a pulse width of <200 fs and a wavelength of 790 nm optimized for Alexa Fluor 350 and Alexa Fluor 594 excitation^22^,^24^. Several objectives were used without digital zoom, 10× (NA 0.3), 20× (NA 0.5), 40× (water immersion NA 0.75), 63× (water immersion NA 1.2). Fluorescence was acquired at a laser line scanning rate of up to 500 Hz and routinely stored as a 256 × 256 × 256 (τ, *x, y*) data cube representing a stack of 8-bit *x-y* images. A short pass 700 nm filter was placed in front of the detector to block out any escaped light from the laser source. Average acquisition times varied between 30–300 s depending on the depth, and the maximum photon count rate was kept well at ~10^5^ s^−1^ to avoid photon pile-up (maximal photon count of the system was near 10^8^ s^−1^).

### TR-FAIM readout and free molecular diffusion: theoretical summary

The speed of rotational and translational components of molecular mobility follows, respectively, the Debye equation 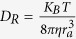 and the Stokes-Einstein equation 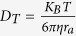, where *D*_*R*_ and *D*_*T*_ are the respective diffusion coefficients, *r*_*a*_ is the hydrodynamic radius, *η* is the medium viscosity, *T* is the absolute temperature and *K*_*B*_ is the Boltzmann constant^19^. These equations predict a linear relationship between *D*_*R*_ and *D*_*T*_: 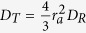. In TR-FAIM, the decay constant of the fluorescence anisotropy time course *r(t*) yields the rotational correlation time *θ* (Fig. 1b) which, in accordance with Perrin’s definitions, has a simple relation to *D*_*R*_: *θ*^−1^ = 6*D*_*R*_. Therefore, the linear relationship in the data indicates that TR-FAIM measurements provide direct reference to both rotational and translational components of molecular mobility.

### Two-photon TR-FAIM: instrumental response calibration and *G*-factor measurements

The instrumental response of the imaging system was extracted by placing a SHG generating surface below the objective; the pulse response was stored as a deconvolution reference for the fluorescence lifetime signals (see below). Fluorescence lifetime was measured, firstly, using a polarizer with the polarization plane parallel to the laser beam polarization; secondly, with a polarizer rotated by 90 degrees (Fig. 1a). The *G*-factor correction reflects a registration error in the system when emission polarization is expected to be uniformly random, i.e., at *t* → ∞: 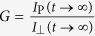, the procedure termed in photonics ‘tail matching’^19^,^20^,^49^. With a duty cycle of 12.5 ns (laser pulsing at 80 MHz), we measured *G* by sampling *I*_P_(*t*) and *I*_⊥_(*t*) at *t* → 12 ns (just before the pulse) in the region of interest containing bath medium only in which diffusion is fast (anisotropy decay *θ* < 1 ns) and unrestricted *a priori*. This yielded *G* = 1.166 ± 0.001 (mean ± SD across the free medium region).

### Two-photon TR-FAIM: Z-factor correction for photobleaching

The host imaging system takes the measurements of *I*_P_(*t*) and *I*_⊥_(*t*) sequentially using a single photomultiplier, to ensure that the instrument response and the detector background noise level are exactly the same for both measurements, especially in the beginning of the 80 MHz duty cycle. Due to the high rate of acquisition, however, indicator molecules are repeatedly exposed to the laser beam, which is likely to result in photobleaching (irreversible loss of fluorescence) for a proportion of the molecules. Because of sequential measurements of *I*_P_(*t*) and *I*_⊥_(*t*), the effect of photobleaching may not be exactly the same for both measurements, thus requiring correction. To provide control measurements for such correction, the measurement sequence for *I*_P_(*t*) and *I*_⊥_(*t*) was set to be ‘symmetrical’: 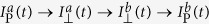. Measurements showed that, throughout the sequence, the effect of photobleaching increases nearly linearly with time (Supplementary Fig. S2c). Denote the relative photobleaching effect on *I*_P_(*t*) over three exposure intervals as


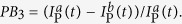


Therefore, to calculate the ‘true’ *r(t*), the average 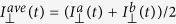 should be corrected by the factor *Z*:


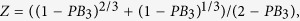


in addition to the G-factor, before relating it to the average 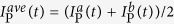.

When we calculated a pixel-by-pixel map of *Z* (Supplementary Fig. S2b) and applied it as a correction factor for *r(t*), we obtained a homogeneous distribution of *r(t*) values in the vicinity of zero at *t* ~ 12 ns (Fig. 2a of the main text), both inside the slice and in the bath (free) medium. This validates the accuracy of the photobleaching correction approach.

To further verify that *r(t*) should indeed approach zero at *t* ~ 12 ns when photobleaching is accounted for, we confirmed this in a separate experiment, by measuring *I*_P_(*t*) and *I*_⊥_(*t) simultaneously*, and therefore under exactly the same photobleaching conditions, using a modified system involving *two* separate photomultipliers (data not shown).

### Preparation

All experiments were carried out in accordance with the national and international rules and regulations for animal experimentation including EU Directive 2010/63/EU of 22 September 2010. All protocols pertinent to animal experimentation were implemented under the corresponding personal and project licenses issued by the UK Home Office. Transverse hippocampal slices, 300–350 μm thick, were obtained from 3–4-week-old mail Sprague-Dawley rats and stored in an oxygenated interface chamber in a perfusion solution containing (mM): 119 NaCl, 2.5 KCl, 0.5 CaCl_2_, 2.5 MgCl_2_ 26.2 NaHCO_3_, 1 NaH_2_PO_4_, 22 glucose, pH 7.4, 305–308 mOsm, for ~1 hour before being transferred to a submersion chamber for imaging experiments, as detailed earlier^22^,^24^. To confirm healthy synaptic activity in the slice before and after TR-FAIM experiments, we routinely recorded field excitatory postsynaptic potentials evoked in area CA1 by electrical stimulation of *stratum radiatum* fibres. To examine extracellular diffusion, we used a water-soluble fluorescence probe Alexa Fluor 350 (hydrazide, sodium salt, Molecular Probes, MW 349; 0.5–1 mM in bath medium).

### Electrophysiology

Whole-cell dye loading of CA3 pyramidal cells was carried out using established patch-clamp routines, as described in detail earlier^22^,^24^,^50^. Acute slices were transferred to the submersion-type recording chamber and superfused, at 34 °C, with artificial cerebrospinal fluid saturated with 95%O_2_/5%CO_2_ containing (in mM): NaCl 125, KCl 2.5, NaH_2_PO_4_ 1.25, NaHCO_3_ 26, glucose 25 (pH7.4; osmolarity 295–305 mOsM) in the presence of 1.3 mM Mg^2+^ and 2.0 mM Ca^2+^. For intracellular measurements, 500 μM AF350 was added to the intracellular solution. The health of patched cells was routinely monitored by testing cell excitability; otherwise, acute slices represented quiescent tissue conditions, with no spontaneous spiking activity in recorded cells.

### Data analysis

Fluorescence recordings were analysed using software developed in-house using MATLAB. Firstly, we established an optimum scale of averaging for individual pixel recordings, to account for inherent microscopic fluctuations in the optical conditions and in the position of the live physiological preparation. A nearest-neighbour radius of ~20 pixels or iso-regional morphological nearest-neighbour (Fig. 2b) was sufficient in most cases to obtain a parameter map (rotational correlation time *θ*) that would provide continuity between shorter (<1 μm) and longer (~10 μm) scales. We thus obtain an anisotropy time course data *r(t*) post-correction (see above) for each pixel as a moving space average. Because the decay of *r(t*) could be unambiguously divided into fast and slow exponential components (Fig. 1e,f), the fitting procedure was relatively straightforward. We therefore used standard MATLAB routines to obtain the best fit for *θ*_*fast*_ and *θ*_*slow*_, pixel-by-pixel, using iterative re-convolution to take into account the finite width of the instrumental response function^51^.

### Statistical methods

The statistical data in graphs are presented as mean ± SEM. We routinely used two-sided *t*-tests to compare sample averages with respect to the null-hypothesis, and for non-Gaussian data scatters the non-parametric Mann-Whitney test was used. These tests, as well as regression analyses, were used as implemented in Origin (Origin Lab Corp). The present study is neither longitudinal, multi-factorial, nor repeated-measures and therefore refers to straightforward statistical inference in cases of one- or two-population comparisons. The source of variance (statistical unit) was considered to be individual preparations, which was equivalent to referring to individual cells or dendritic spine structures: one spine structure was sampled per one cell per one brain slice. In total, 36 Sprague-Dawley rats were used for all measurements.

## Additional Information

**How to cite this article:** Zheng, K. *et al*. Nanoscale diffusion in the synaptic cleft and beyond measured with time-resolved fluorescence anisotropy imaging. *Sci. Rep.* **7**, 42022; doi: 10.1038/srep42022 (2017).

**Publisher's note:** Springer Nature remains neutral with regard to jurisdictional claims in published maps and institutional affiliations.

## Supplementary Material

Supplementary Information
