# Supplementary material for: Nanoscale diffusion in the synaptic cleft and beyond measured with time-resolved fluorescence anisotropy imaging
Source: Sci Rep. 2017 Feb 9;7:42022. doi: 10.1038/srep42022 (PMC5299514; doi:10.1038/srep42022)
Supplement: Supplementary Information [file srep42022-s1.pdf]

Nanoscale diffusion in the synaptic cleft and beyond measured with time-resolved fluorescence anisotropy

Kaiyu Zheng[1], Thomas P. Jensen[1], Leonid P. Savtchenko[1,2], James A. Levitt[3], Klaus Suhling[3], Dmitri A. Rusakov[1]

[1] Institute of Neurology, University College London, Queen Square, London WC1N 3BG, UK;

[2] Institute of Neuroscience, University of Nizhny Novgorod, 603950 Nizhny Novgorod, Russia;

[3] Department of Physics, King's College London, Strand, London WC2R 2LS, UK.

*Correspondence and requests for materials should be addressed to: D.A.R (email: d.rusakov@ucl.ac.uk) or K.Z. (email: k.zheng@ucl.ac.uk).

Supplementary information

Unit: ns	Free medium	Extracellular			Intracellular		
		CA1 neuropil	CA3 neuropil	Cleft	Soma	Branch	Spines
θ_{fast}	0.174	0.235	0.271	0.297	0.29	0.392	0.292
S.D.	0.028	0.042	0.06	0.086	0.027	0.11	0.055
θ_{slow}	n/a	7.27	11.5	12	1.22	4.24	1.48
S.D.	n/a	1.9	15	6.5	0.73	11	0.36
% slow (range)	0	0.5-3%			3-20%		

Supplementary Table S1. Average values and the partial weight of slow versus fast anisotropy decay components (standard double-exponent fitting), across the experimental areas of interest in acute hippocampal slices.

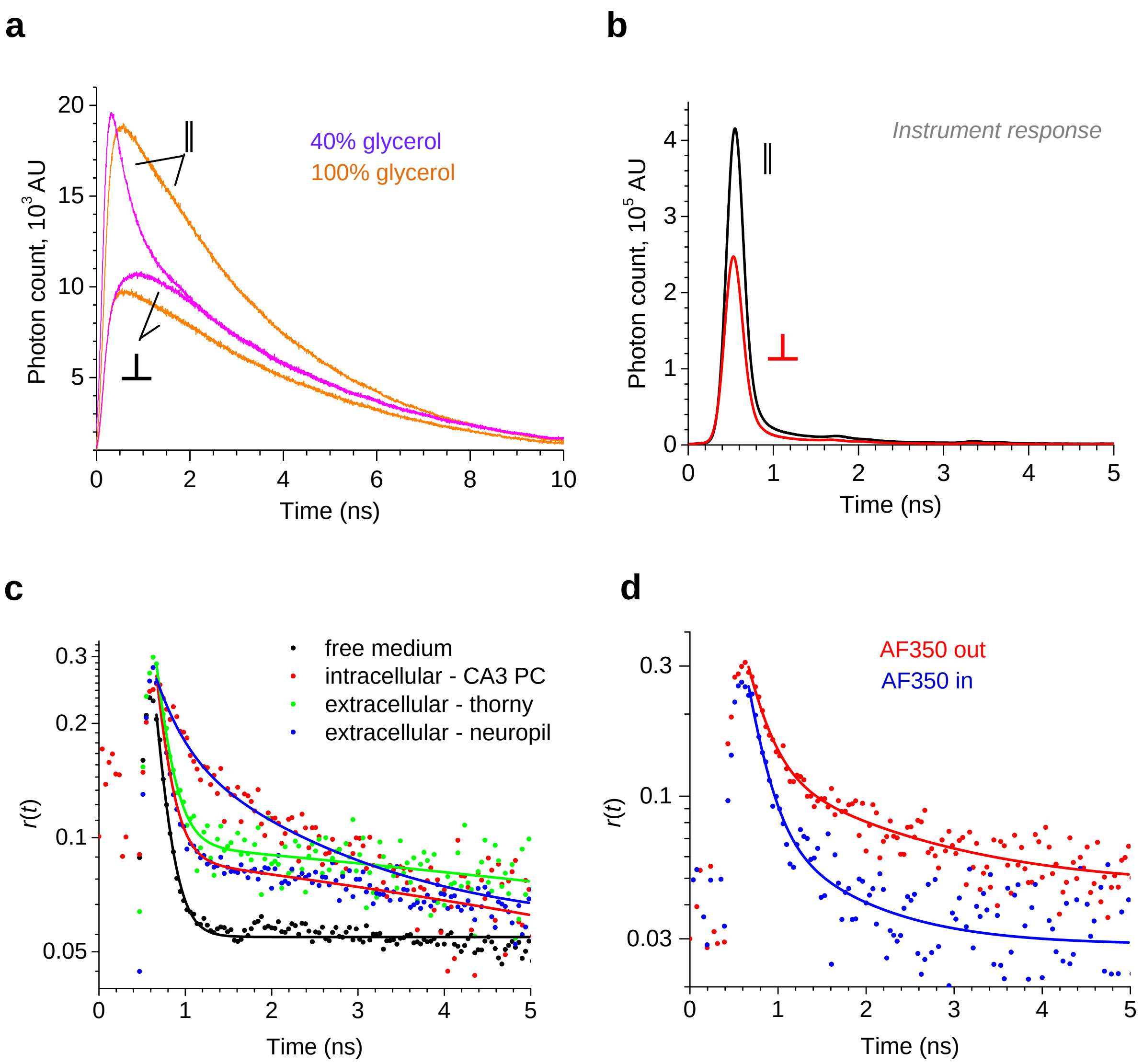

Supplementary Fig. S1. TR-FAIM measurements provide unbiased readout of molecular diffusivity in conditions of organized brain tissue.

(a) Example of the AF350 fluorescence decay dynamics recorded in the two polarization detectors (parallel and perpendicular) in solutions of different viscosity, as indicated.

(b) Example of the instrument response function recorded in the two-detector FLIM system, as indicated.

(c) Examples of the AF350 fluorescence anisotropy decay for different regions of interest in a hippocampal slice (and free medium), as indicated.

(d) Washout of bath-applied AF350 increases the relative contribution of its slow fluorescence anisotropy decay component many-fold, as indicated, which reflect the increased fraction of immobile (membrane-bound) compared to free-diffusing molecules in the extracellular space. One-experiment example, average data for the CA1 slice area.

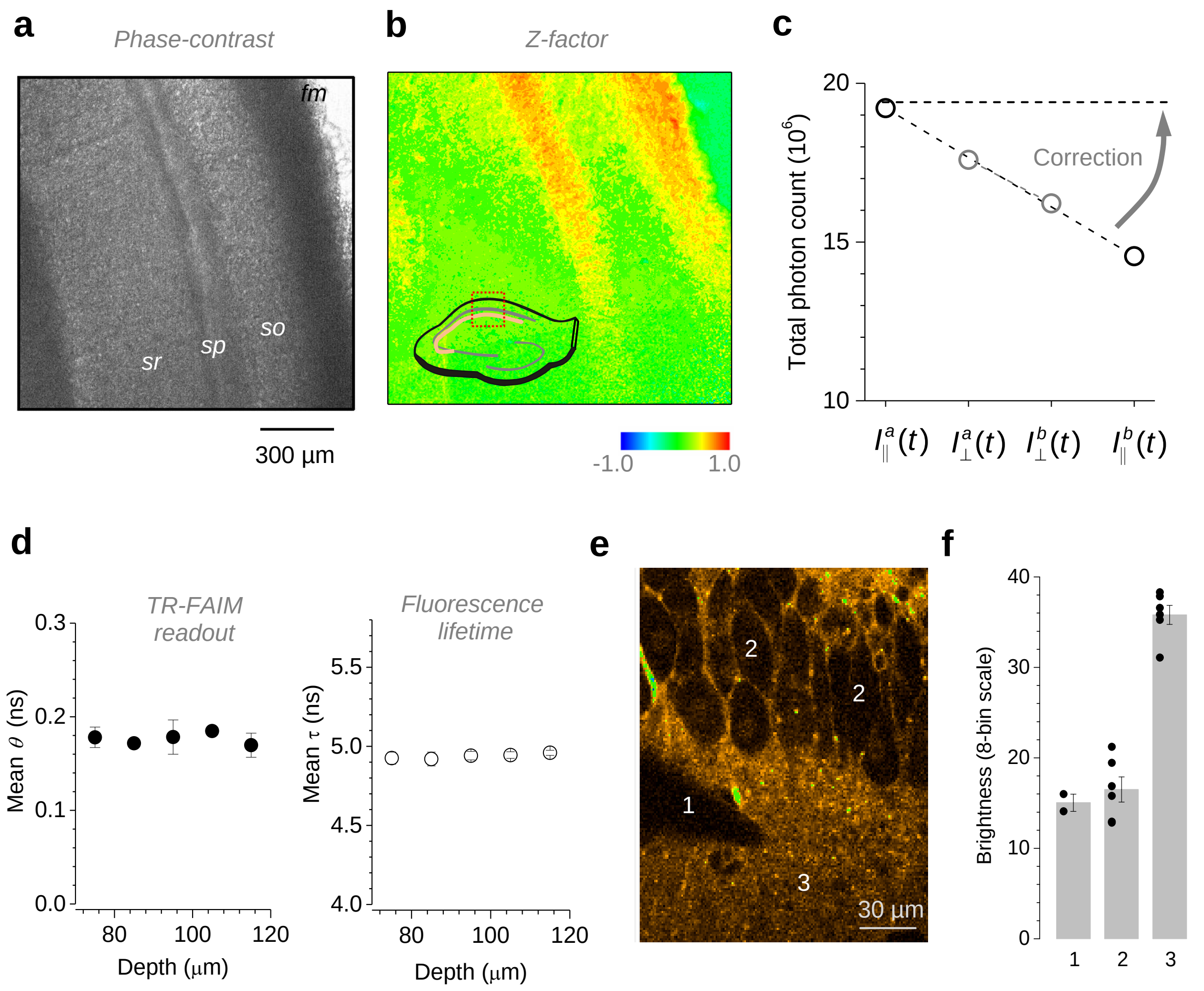

Supplementary Fig. S2. TR-FAIM measurements provide unbiased readout of molecular diffusivity in conditions of organized brain tissue.

(a) A phase-contrast image of the hippocampal slice (*sr, stratum radiatum; sp, stratum pyramidale; so, stratum oriens; fm,* free medium) prepared for extracellular dye imaging.

Inset in b depicts the region of interest.

(b) Mapping the effect of AF350 photobleaching on TR-FAIM readout $r(t)$, pixel-by-pixel, as Z-factor (Methods) in sequential measurements of $I_{\parallel}(t)$ and $I_{\perp}(t)$; acute brain slice fragment shown in (a); also illustrated in the inset.

(c) The average effect of AF350 photobleaching (dots, measured values) in four sequential image acquisitions (abscissa) for the two detectors registering $I_{\parallel}(t)$ and $I_{\perp}(t)$ signals, as indicated. Dotted line, the average effect of photobleaching showing clear linear regression with time; the curved arrow points to the adjustment required to compensate for the effect of photobleaching. See Methods for technical detail.

(d) Light scattering in brain tissue has no effect on the fluorescence anisotropy decay or the fluorescence lifetime. The value of θ (fast rotational correlation time, left) and τ (fluorescence lifetime, right) for AF350 obtained at different depths in acute hippocampal slices, as indicated. Measurements are averaged for n = 6 regions of interest in two slices (mean ± s.e.m.).

(e) Estimating residual intracellular fluorescence in acute slices perfused with a cell-impermeable Alexa Fluor indicator: a 2PE image (λ^{2p}_{x} = 810 nm; false colors) of CA1 area, ~100 µm deep. Focal fluorescence due to 2PE (within a ~1 µm optical layer) was averaged: within the pipette void (1, background signal), in the pyramidal cell soma lumen (2), and in the synaptic neuropil (3; includes ~20% of the extracellular space), as indicated.

(f) Average fluorescence intensity (mean ± s.e.m., eight-bin grey scale) for the ROIs shown in (e): 15.0 ± 1.0 (1, neuropil), 16.5 ± 1.4 (2, background noise), and 35.8 ± 1.1 (n = 6 slices). Denote F_e and F_i as fluorescence signals originating from, respectively, the extracellular space and the intracellular lumen. Because the extracellular volume fraction in CA1 area is ~20%, fluorescence in the neuropil (area 3) represents 0.2F_e plus 0.8F_i plus background signal (area 1) whereas fluorescence in the lumen is F_i plus background. Thus, the following relationship holds: $(0.2F_e + 0.8F_i)/F_i$ = (35.8-15)/(16.5-15), which yields F_e/F_i=0.015. This estimate implies that the intracellular signal intensity is ~1.5% of extracellular signal intensity, which should correspond to ~6% of total fluorescence in the neuropil containing ~20% extracellular volume fraction.

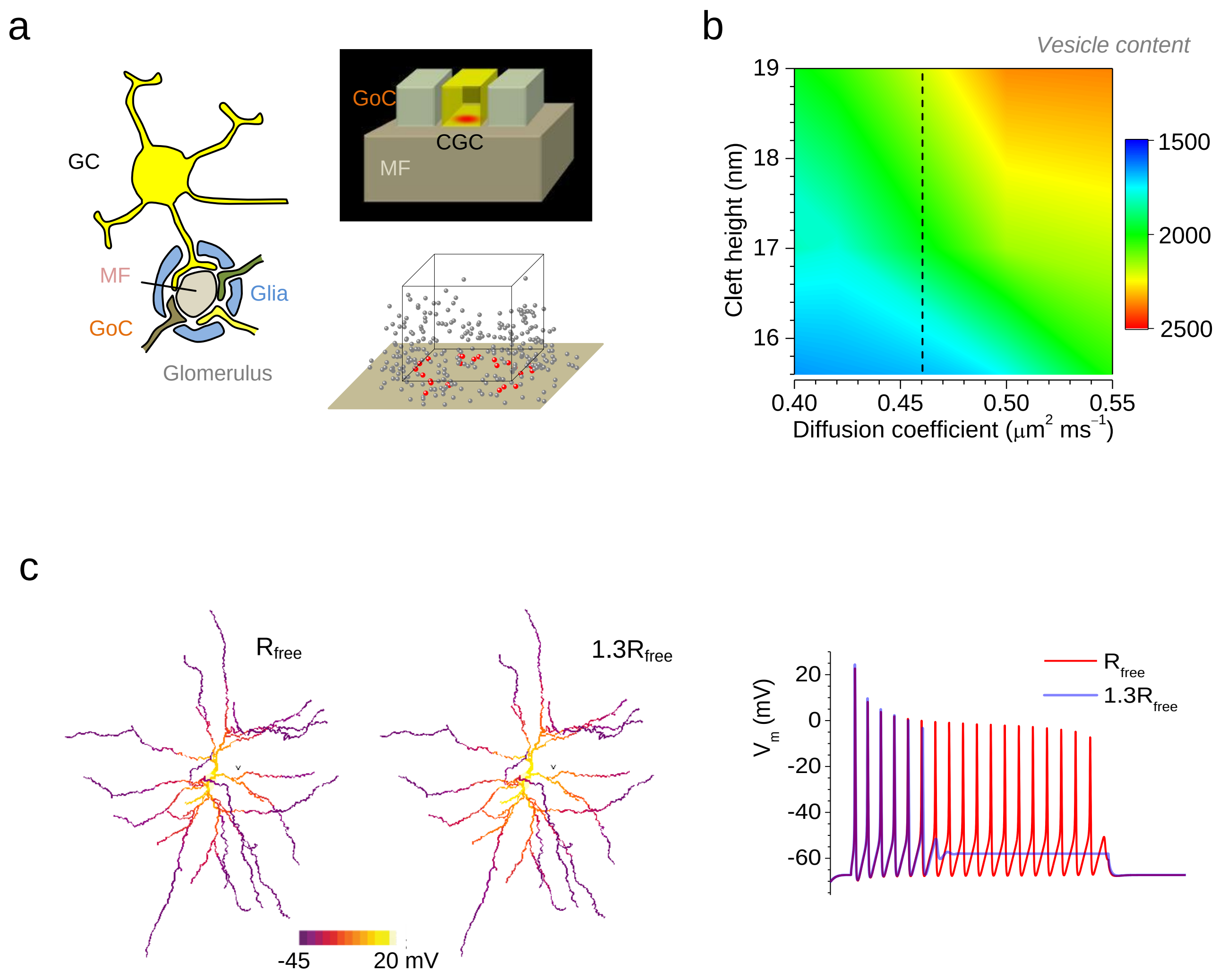

Supplementary Fig. S3. Measurements of free nanoscale diffusion with TR-FAIM provide important constrains for evaluating basic features of neural signaling.

(a) *Left,* Schematic depicting the cerebellar synaptic circuit explored earlier in Monte Carlo modeling tests [1]: GC, granule cells; MF, mossy fiber; GoC, golgi cell. *Right*, model geometry representing the MF-CG synapse (top) and a snapshot of glutamate diffusion 2 ms post-release (bottom, 2500 molecules released, half of molecules are depicted for clarity).

(b) Experiment-based evaluation of vesicular glutamate content (number of molecules) at cerebellar mossy fiber -granule cell synapses (false color scale) constrained by the

plausible range of synaptic cleft heights (ordinate) and intra-cleft glutamate diffusion coefficient (abscissa), as described in detail previously [1]. Dotted line corresponds to the diffusivity estimate obtained presently, which critically constrains the evaluation.

(c) Extracellular medium resistance affects neuronal excitability: theoretical assessment. Left**,** membrane potential distribution (color-coded) during the somatic spike in the typical reconstructed nerve cell taken from the experimental library (layer 5 interneuron C050800E2, NeuroMorpho database / www.neuromorpho.org), with extracellular resistivity either equal to that of a free medium (R_{free} =59 Ohm·cm) or increased by 30% to account for the extracellular diffusion retardation measured here, as indicated; Right, a 30% increase in R_{free} dampens cell excitability curtailing depolarization-induced spiking (red and blue).

Supplementary References

1 Savtchenko, L. P., Sylantyev, S. & Rusakov, D. A. Central synapses release a resource-efficient amount of glutamate. *Nat Neurosci* **16**, 10-12 (2013).
